# Perspectives on Clinical Adoption Barriers to Blood-Based Multi-Cancer Early Detection Tests across Stakeholders

**DOI:** 10.3390/jpm14060593

**Published:** 2024-06-01

**Authors:** Monica M. Schroll, Elissa Quinn, Daryl Pritchard, Allina Chang, Kristen Garner Amanti, Omar Perez, Arushi Agarwal, Gary Gustavsen

**Affiliations:** 1 Health Advances LLC, 101 2nd Street, Suite 800, San Francisco, CA 94105, USAggustavsen@healthadvances.com (G.G.); 2 AstraZeneca, Wilmington, DE 19803, USA; elissa.quinn@astrazeneca.com (E.Q.); omar.perez@astrazeneca.com (O.P.); 3 Personalized Medicine Coalition, Washington, DC 20036, USA; dpritchard@personalizedmedicinecoalition.org

**Keywords:** blood-based multi-cancer early detection tests, MCED tests, healthcare providers, patients, payers, personalized medicine adoption, implementation, survey

## Abstract

Current United States Preventive Services Task Force (USPSTF) recommendations include routine screening for breast, cervical, colorectal, and lung cancer; however, two out of every three cancer cases occur in other indications, leading to diagnoses in advanced stages of the disease and a higher likelihood of mortality. Blood-based multi-cancer early detection (MCED) tests can impact cancer screening and early detection by monitoring for multiple different cancer types at once, including indications where screening is not performed routinely today. We conducted a survey amongst healthcare providers (HCPs), payers, and patients within the U.S. health system to understand the current utilization of cancer screening tests and the anticipated barriers to widespread adoption of blood-based MCED tests. The results indicated that the community favors the adoption of blood-based MCED tests and that there is broad agreement on the value proposition. Despite this recognition, the survey highlighted that there is limited use today due to the perceived lack of clinical accuracy and utility data, high out-of-pocket patient costs, and lack of payer coverage. To overcome the hurdles for future widespread adoption of blood-based MCED tests, increased investment in data generation, education, and implementation of logistical support for HCPs will be critical.

## 1. Introduction

Cancer remains the second leading cause of death [1] in the United States and a significant challenge for global health. Cancer mortality rates have declined in the US over the past three decades [2], in part due to the implementation of cancer screening and early detection tests into routine practice for some highly prevalent cancers [3]. Nonetheless, it is estimated that in 2023 there were ~1,960,000 incident cancer cases and ~610,000 cancer deaths in the United States alone [2].

Cancer screening and earlier detection are pivotal to guiding interventions earlier in asymptomatic cancer patients [4], often resulting in improved five-year survival rates and quality-of-life measures, as well as potential significant reductions in the cost and complexity of cancer care [5,6,7]. The United States Preventive Services Task Force (USPSTF), which is responsible for releasing evidence-based recommendations to improve population health, currently recommends routine screening for breast, cervical, colorectal, and lung cancer [8]. However, two out of every three cancer cases occur in cancer indications without recommended routine screening, leading to diagnoses in advanced stages of the disease and a higher likelihood of mortality [9].

Historically, new cancer screening tests are not widely adopted into practice until they are recommended within the USPSTF guidelines. Once a cancer screening test is included in the USPSTF guidelines with a grade “A/B” rating, the Patient Population and Affordable Care Act (ACA) requires coverage of the test without cost sharing [10]. For many cancer screening and early detection tests, such as Cologuard (Exact Sciences) for colorectal cancer screening, this leads to a significant uptake in lives covered and test usage [11].

Current recommended cancer screening and early detection tests, such as colonoscopies, mammograms, low-dose CT scans, and cervical cytology, also face several hurdles hindering equitable access in a real-world setting. Barriers to adoption include low screening compliance rates among eligible populations, low test sensitivity for early-stage disease, high false positive rates, and equivocal cost-effectiveness [12]. Insufficient compliance with, or access to, reliable early screening technologies leads to cancer diagnoses at later stages. Such diagnoses not only pose greater challenges for effective treatment [13,14], but also have negative economic implications for the patient and their families [15,16,17].

The next frontier for cancer screening involves multi-cancer detection (MCD) tests, also referred to as multi-cancer early detection (MCED) tests. These tests are poised to redefine the current landscape by screening for multiple different cancer types at once and detecting cancers that would otherwise have been missed by traditional routinely recommended screening tests [18,19]. The predominant technologies for multi-cancer screening tests include whole-body MRI scans [20] and blood-based liquid biopsies [21]. Future MCED technologies are expected to become available using breath, urine, saliva and stool samples [22].

Blood-based liquid biopsies rely on circulating tumor DNA shed by early-stage tumors [23]. Blood-based liquid biopsies have been available for decades and are used post-cancer diagnosis as companion diagnostics for identifying genetic mutations, assisting in treatment decisions, and predicting outcomes; examples include the cobas^®^ EGFR Mutation Test for NSCLC [24], Guardant360^®^ CDx [25], FoundationOne Liquid CDx [26], and the CellSearch Circulating Tumor Cell Test [27]. Blood-based liquid biopsy tests for multi-cancer detection are the focus of this research. Commercially available blood-based MCED tests include Galleri (GRAIL) and OneTest [28], with several in development, including Cancerguard (Exact Sciences) [29], CancerSEEK [30], and others [31,32,33,34]. MCED tests are typically developed to convey high specificity and variable sensitivity, which translates to low false positive rates, typically with the compromise of high false negative rates. This can help to avoid overdiagnosis at a population level, but represents the converse strategy of traditional cancer screening tests which are typically optimized for high sensitivity for initial screening and are complemented by follow-up confirmatory testing [35,36].

Given their nascency and lack of reimbursement, the role of blood-based MCED tests are not commonly employed in clinical practice today, and their overall role in patient care is still undefined [37]. To help facilitate the appropriate integration of MCED tests into patient care, it is important to understand the perspectives of health care providers (HCPs), payers, and patients on the anticipated challenges to implementation. A survey of a representative cohort of U.S. HCPs, payers, and patients was conducted to gather community perspectives on barriers to broad adoption of MCED tests. Compiling viewpoints across key stakeholder groups allows for a better understanding of implementation challenges and will help inform the development of clinical access strategies.

## 2. Methods

Survey development was informed by 60 min qualitative phone interviews with primary care providers (*n* = 2), OB-GYNs (*n* = 2), oncologists (*n* = 1), pulmonologists (*n* = 1), and payers (*n* = 2) regarding the current adoption of cancer prevention and early detection tests, the awareness and adoption of MCED tests, and the barriers to MCED test adoption. The survey development interviews revealed that primary care providers and OB-GYNs were the key target specialty to understand the barriers to MCED test adoption for this study and therefore were the only HCPs included in the survey.

Three separate surveys of representative cohorts of U.S. HCPs (*n* = 238) (primary care providers and obstetrics and gynecologists (OB-GYNs)), payers (*n* = 40), and patients (*n* = 116), were conducted to evaluate the current cancer screening and early detection landscape and gather perspectives on barriers to implementation of new cancer screening technologies. The survey respondents were recruited to take an online survey by a market research insight collection company (GRG Health) from June–July 2023 in a double-blinded fashion so that the identity of the respondent was not revealed to us (the sponsor), and the sponsor of the study was not revealed to the respondent. If participants met the screening criteria (below), they proceeded to complete a 20 min questionnaire (Supplementary Methods) and received an $75–100 honorarium. Respondent demographics are displayed in Table 1.

Primary Care Providers and OB-GYNs. Respondents were required to have been in practice between 3 to 35 years, to spend the majority of their time in direct patient care, to see an average of at least 30 patients per month, and to be familiar with at least one cancer screening and early detection test. These criteria for selection were to ensure that respondents were active in patient care and would be able to provide context on historical experience with cancer screening and early detection tests and be able to consider the future adoption challenges of MCED tests.

Payers. Respondents were required to hold one of the following titles: Medical Director, Clinical Advisor, Chief Medical Officer, Laboratory Benefits Manager, or Oncology Services Manager. In addition, payer respondents needed to have at least 2 years of experience at a payer organization that covers at least 10,000 lives. Respondents were required to either be frequently, or directly, involved in medical policy and coverage decisions relating to diagnostic tests for oncology. These criteria were enforced to ensure that respondents would have historical context and knowledge of oncology diagnostic test coverage decisions and be able to speak to requirements and challenges for extending coverage to MCED tests over a mix of insurance plans.

Patients. Respondents were required to be between the ages of 30 and 75, to never have received a previous cancer diagnosis, and to understand the future barriers to receiving MCED tests. These selection criteria are considered the aspirational population for MCED tests, and therefore including these respondents in the survey allowed for understanding of potential drivers and barriers to adoption in a wide population.

## 3. Results

### 3.1. HCP and Payer Cancer Screening and Early Detection Testing Current Landscape

**Figure 1:**
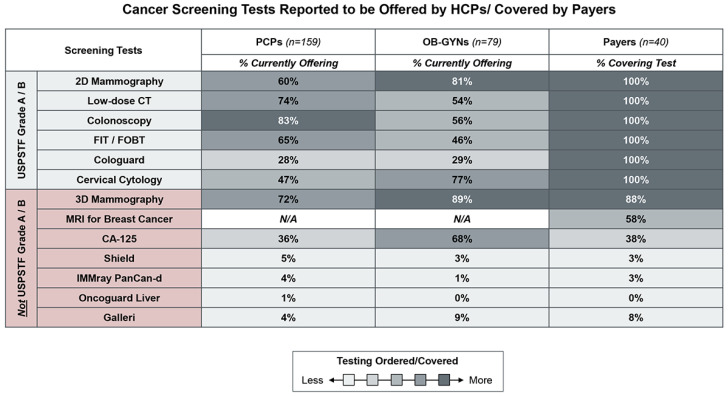
Cancer screening tests reported to be offered by HCPs/covered by payers. Survey respondents were asked which cancer screening tests they currently offer in their practice or are covered by their plans for HCPs and payers, respectively. The tests inquired about included those tests with USPSTF Grade A/B ratings, including 2D mammography, low-dose computed tomography (CT), colonoscopy, fecal immunochemical test/fecal occult blood test (FIT/FOBT), Cologuard, and cervical cytology and a sampling of tests without USPSTF Grade A/B ratings, including 3D mammography, MRI for breast cancer, cancer antigen-125 (CA-125), Shield (i.e., blood-based liquid biopsy for colorectal cancer), IMMray PanCan-D (i.e., blood-based liquid biopsy for pancreatic cancer), Oncoguard Liver (i.e., blood-based liquid biopsy for hepatocellular cancer) and Galleri (i.e., blood-based liquid biopsy for multi-cancer early detection). Note that all payers are required to cover cancer screening tests with USPSTF Grade A/B ratings < 10% of HCPs and payers reported to offer/cover available blood-based liquid biopsy tests today.

HCPs reported offering breast, cervical, colorectal, and lung cancer screening tests most commonly, which are the indications with grade A or B ratings by USPSTF. In addition, HCPs reported frequently offering cancer screening tests that are typically reimbursed by payers. These tests either have very low costs (e.g., cancer antigen (CA)-125), or have strong evidence of clinical utility data and are on track to receive grade A/B ratings in the future (e.g., 3D mammography and breast MRI). There is limited HCP familiarity and payer coverage of marketed blood-based cancer screening tests (Figure 1).

**Figure 2:**
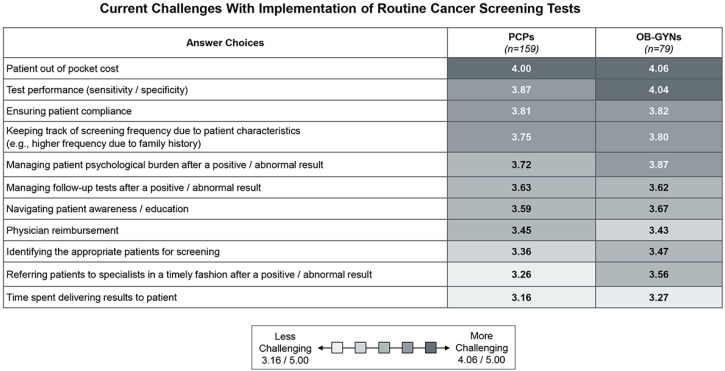
Current challenges with implementation of routine cancer screening tests. When asked to rate the current challenges with the implementation of routine cancer screening where 1 is not challenging and 5 is very challenging: primary care providers rated all answer choices provided between 3.16–4.00, and OB-GYNs rated all answer choices provided between 3.27–4.06. Both HCP types rated patient out-of-pocket cost as the most challenging aspect to implementation today.

Of note, HCPs and patients reported that the patient out-of-pocket (OOP) cost of current cancer screening tests is the most challenging factor today for implementing routine cancer screening tests, even those with a USPSTF grade A/B, such as colonoscopies and mammograms (Figure 2). In addition to patient OOP cost, HCPs viewed test performance (sensitivity/specificity), ensuring patient compliance, and keeping track of screening frequency due to patient characteristics (e.g., higher frequency due to family history) as the next most challenging factors with implementing routine cancer screening tests (Figure 2).

### 3.2. Patient Cancer Screening and Early Detection Testing Current Landscape

Of the patients surveyed, 88% of all patients who are recommended a cancer screening test by an HCP reported following through with receiving the test (Figure S1), which is higher than the national reported averages by the CDC [38]. Analyzing the surveyed patient responses by socio-economic status, 100% of patients with an annual household income > $75,000 reported that they always obtain their recommended screening, whereas only 80% of patients with an annual household income < $75,000 reported that they follow through with recommended screening (Figure S1).

Patients who followed through with receiving an HCP recommended screening test reported doing so primarily due to their understanding of the importance of cancer screening to their health and reminders from their HCPs (Figure S2). The patients who did not follow through with HCP recommended cancer screening ranked fear of a cancer diagnosis, not having transportation to the test site/test site being too far away, and no family history of cancer as the top reasons (Figure S2).

Overall, patients reported that receiving screening is not considered difficult, rating the overall difficulty of completing the test as a 2.0/5 with 5 being extremely difficult and 1 being not at all difficult (Figure S3). Nonetheless, patients ranked non-invasive testing options and more convenient testing locations as the top ways to improve current cancer screening tests (Figure S3).

### 3.3. Factors That Influence Adoption of New Cancer Screening and Early Detection Tests

HCPs and payers recognized the need for more cancer screening options. HCPs ranked ovarian, thyroid, and pancreatic cancer as the top three indications where they are most interested in additional screening options (Figure S4). HCPs were willing to incorporate novel cancer screening and early detection tests into practice prior to recommendation by the USPSTF guidelines if they are supported by clinical data and are screening for a cancer type with high unmet need (Figure S5). HCPs ranked evidence of test accuracy and clinical utility as the most important factors for adopting new cancer screening and early detection tests prior to their inclusion in USPSTF guidelines (Figure S6). Better patient outcomes and the ability to make informed clinical decisions are considered the most compelling clinical utility data types (Figure S6).

When surveyed, payers expressed that the total cost of cancer care is an ongoing challenge for the health system (rated 3.8/5) and that cancer care represents ~20% of average insurance plan spend today (Figure S7). Beyond cheaper and more efficacious drugs, payers reported that the best way to reduce the cost of cancer is by providing more tools for earlier cancer detection (Figure S7). Specifically, similar to HCPs, payers ranked pancreatic, ovarian, and bladder cancer as the top three indications where screening is not currently endorsed and where an early detection test would reduce the cost of care (Figure S4).

### 3.4. Stakeholder Perceptions of Blood-Based MCED Tests

**Figure 3:**
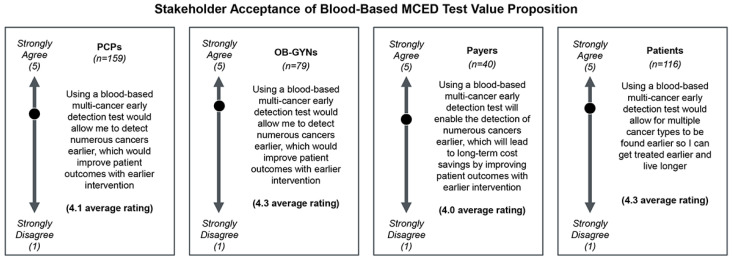
Stakeholder acceptance of blood-based MCED test value proposition. When asked to rate if the respondent agrees with the following statements (see figure for verbatim statement presented to each stakeholder), on a scale of 1 to 5, where 1 is strongly disagree and 5 is strongly agree, the average rating across all stakeholders was >4, indicating strong agreement with the value proposition of MCED tests.

Of the surveyed HCPs and payers, >90% and 100%, respectively, reported being aware of MCED tests (Figure S8). Additionally, all surveyed stakeholders perceive blood-based MCED tests as having a strong value proposition, as defined in Figure 3.

However, despite high awareness and recognition of the value proposition, <10% of HCPs reported having ordered an MCED test, and 80% of payers had not yet evaluated an MCED test for coverage under their plan at the time of the survey (Figure S8).

The majority of patients also expressed excitement and likelihood to seek out blood-based MCED testing. Of the surveyed patients, only 12% reported that they were unlikely to seek out MCED testing (Figure S9). Additionally, surveyed patients indicated that they would be more motivated to receive a blood-based MCED test if their HCP dedicated time to discuss the advantages and disadvantages of the blood-based MCEDs and answer their questions (Figures S10 and S11).

**Figure 4:**
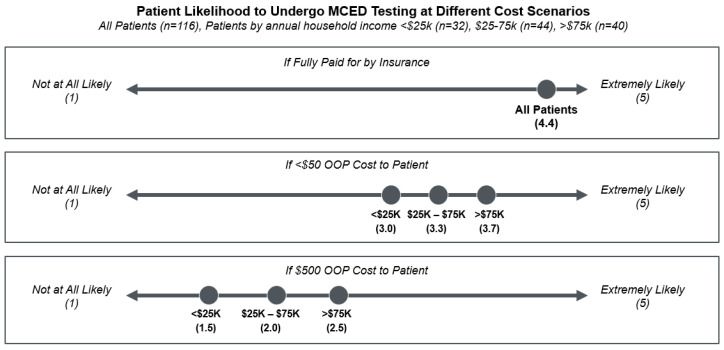
Patient likelihood to undergo MCED testing: Patients were asked on a scale of 1 to 5, where 1 is not at all likely and 5 is extremely likely, how likely they were to undergo MCED testing with different cost scenarios. When the cost is fully covered by insurance providers, patients were mostly likely to undergo MCED testing. When the cost is <$50 or $500, likelihood decreases, with patients with a lower annual household income being less likely than those with higher annual household incomes. Note: OOP = out of pocket.

When factoring in the potential OOP cost, patients’ likelihood of choosing to undergo a blood-based MCED test decreased as a function of cost and socio-economic status (Figure 4). The patient survey illustrated that if MCED testing was fully paid for by insurance, patients are most likely to choose the test (rated 4.4/5), and as out-of-pocket cost increases, the likelihood to choose the tests decreases in all patients, with the largest decrease in patients with an annual household income < $25,000 (Figure 4).

### 3.5. Anticipated Challenges to MCED Test Adoption

**Figure 5:**
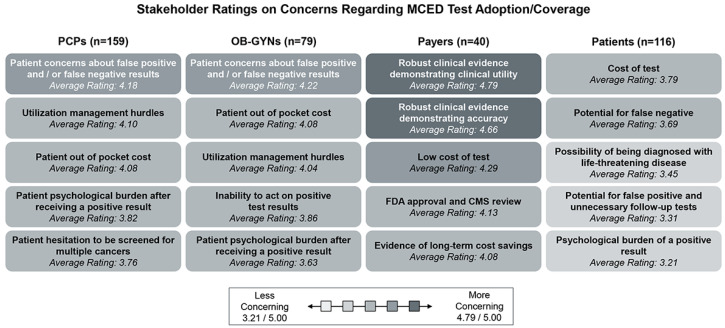
Stakeholder ratings on concerns regarding MCED test adoption/coverage: Surveyed stakeholders were asked to rate how concerning the above answer choices (see figure) were when considering MCED test adoption/coverage. Responses are listed in order of most to least challenging and shaded grey based on the legend from 3.21/5.00–4.79/5.00. Note: FDA = Food and Drug Administration, CMS = Centers for Medicare and Medicaid Services.

HCPs, both primary care providers and OB-GYNs, rated patient concerns about false positive and/or false negative results, payer utilization management policy challenges, and patient OOP costs as the top three concerns when intending to adopt MCED tests. Patients rated the cost of the test, the potential for false negatives, and the possibility of being diagnosed with a life-threatening disease as their top three issues impeding their use of blood-based MCED tests. Payers reported that robust clinical evidence demonstrating accuracy and utility is the most important factor needed to extend coverage to MCED tests. All survey answer choices were rated >3.0/5.0, suggesting that all of the anticipated concerns to overcome for MCED test adoption may have high impact (Figure 5). When assessing how stakeholders view the significance of the anticipated barriers, payers considered the barriers as the most significant.

## 4. Discussion

Blood-based MCED tests have the potential to revolutionize cancer screening and significantly improve patient outcomes [18,19]. The convenience and ease of a blood draw at a range of sites would improve access to routine cancer screening for many patients. Once clinically validated and adopted into practice, blood-based MCED tests hold the promise of simultaneously screening for an array of cancers, some of which do not have routine screening today but are associated with considerable mortality and morbidity, such as pancreatic and ovarian cancer.

The survey of HCPs, payers, and patients in the United States indicated that there is an appetite throughout the community to adopt blood-based MCED tests, and there is agreement that these tests have the potential to save lives. Despite broad recognition of the value of blood-based MCEDs, the study highlighted that there is limited use and coverage today due to a myriad of factors.

The survey results indicated that HCPs’ baseline requirement to adopt blood-based MCED tests into clinical practice is validity data that support test accuracy, characterized by sensitivity, specificity, positive predictive value (PPV), and negative predictive value (NPV) [39]. Although predictive diagnostic tests can never be expected to achieve 100% sensitivity and specificity, current blood-based MCED tests are usually associated with lower false positive rates and higher false negative rates than current USPSTF recommended cancer screening tests. While this may help to avoid overdiagnosis, it could create confusion and hesitation for patients and providers alike [40].

Blood-based MCED test manufacturers should focus on producing clinical accuracy data that garner incorporation into clinical guideline recommendations endorsed by leading medical and professional societies. Additionally, stakeholders within the blood-based MCED community (manufacturers, the United States Food and Drug Administration, consortiums, health systems, and health plans) should provide HCP- and patient-targeted educational resources on false positives/false negatives and related risks in patient care. Educating HCPs and equipping them with the ability to educate their patients on the accuracy of blood-based MCED tests and the implications of a false positive/negative result should help increase appropriate test usage.

Widespread adoption and payer coverage of blood-based MCED tests hinge on clinical utility data showing that use of blood-based MCED tests can improve patient management and net health outcomes [40]. The survey results showed that any OOP cost is a key barrier for patients. Lack of payer coverage will likely continue to hinder blood-based MCED test adoption until payers have health outcome data that demonstrate clinical utility.

Proving the clinical utility of blood-based MCED tests in higher-risk populations by using intermediate or surrogate outcome measures standardized by various organizations assessing quality measures, such as the MCED and BLOODPAC Consortiums [40,41] and the broader medical community, would likely expedite the path to adoption and reimbursement.

The survey results highlighted that payers and HCPs are most interested in attaining clinical utility data in patients with clinical factors that are perceived to be more appropriate for blood-based MCED testing, specifically patients over age 65 with a family history of cancer or genetic predispositions to cancer. Adoption and payer coverage may then expand to lower-risk populations over time. A precedent for this strategy may be the use of noninvasive prenatal tests (NIPT for congenital abnormalities in fetal DNA). NIPT was first launched in smaller, high-risk populations of women with increased risk for fetal aneuploidy [42], then made available to a broader, average-risk population after data supported usage for additional populations [43]. Another way to reach widespread adoption of blood-based MCED tests may be to first focus on specific tumor types where screening tests are reported to have higher impact (Figure 1), such as CA-125 for ovarian cancer, and low-dose computed tomography (CT) for lung cancer, or where there is heightened interest from the community due to an urgent unmet need, such as in pancreatic cancer.

Collaborations between blood-based MCED test manufacturers, health systems, and/or payer organizations can help to generate near-term clinical utility data and accelerate knowledge generation. For example, GRAIL and Point32Health (a nonprofit health and well-being organization) are conducting a multi-phased pilot in which Point32Health is providing Galleri to certain employees (depending on age and family history), as well as HCP groups serving Point32Health’s commercial members in order to generate real-world evidence and assess the impact on care resource utilization and other outcomes [44]. This type of evidence generation may increase the speed at which MCED tests are reimbursed and available to patients across the United States.

To seamlessly integrate blood-based MCED tests into clinical practice, HCPs need a testing process that is built into the patient visit workflow and clear guidance for testing practices. There are multiple stakeholders that can deploy thoughtfully designed strategies to attenuate these issues.

Different organizations, such as the MCED Consortium and BLOODPAC Consortium, are developing recommendations for baseline implementation of MCED testing, including standardized procedures for use in a clinical setting and communication of blood-based MCED results [22,35,40,41]. Continued support for these recommendation-building efforts by the medical community, patient advocacy groups and industry leaders may help to improve practice guidelines and raise awareness of MCED test utility.

Industry stakeholders can also support broader HCP adoption of blood-based MCED tests by helping to develop and provide access to credible, impartial educational material describing blood-based MCED tests and including updated clinical data and recommendations related to patient eligibility and testing frequency.

Finally, health systems can further advance MCED test adoption through improved system-level education, workflow implementation tools, and defined pathways and algorithms for screening appropriate individuals at the point of care. EMR notifications, decision support tools, and results interpretation assistance for blood-based MCED tests can help minimize workforce burden. Trained screening navigators equipped to work alongside physicians and patients throughout the diagnostic process, enabling expeditious follow-up on test results, financial literacy, compassionate ‘hand-holding’, and effective patient-centric communication, will improve patient adherence to downstream interventions while relieving physician burden.

The survey study was subject to several limitations. While the cohorts surveyed were representative of the U.S. HCP, payer, and patient populations, the sample size may limit the result’s generalizability to the entire U.S. stakeholder population. The sample size also restricts the ability to analyze statistical significance across stakeholder survey answers. Additionally, specific demographics of patients may be over-represented, including 55% of participants being <50 years old and 66% of participants identifying as white, or may be under-represented, including only 6% of participants identifying as Latin American/Hispanic. Additionally, the results reported by the survey respondents may not reflect the current NCCN or USPSTF guideline recommendations for cancer screening; an example of this is the high use of CA-125, which is not a NCCN/USPSTF recommended cancer screening test.

## 5. Conclusions

In conclusion, the findings of this study can help build community understanding of these anticipated barriers and inform strategies to alleviate them and build a foundation for clinical adoption of blood-based MCED tests. In order to improve payer coverage and increase adoption, supporting clinical data and widespread educational efforts may be needed to leverage a multi-stakeholder approach to knowledge acceleration. The future direction of our research is to continue to survey the medical community to better understand actionable steps to alleviate the barriers discovered in this study. Understanding the next best actions for the barriers and working with the medical community will accelerate the opportunity for blood-based MCED tests to shift the current cancer screening paradigm by identifying cancer earlier and ultimately improving patient outcomes. In order to harness the true potential of blood-based MCED tests, continued collaborative effort by the medical community and all other involved stakeholders is critical.

## Figures and Tables

**Table 1:** Demographics of all survey respondents delineated by stakeholder types. Stakeholders for this study include health care providers comprising general practitioners (primary care/family medicine/internal medicine) and obstetrics and gynecologists (OB-GYN), payers, and patients. All respondents were 21 years of age or older at the time of the survey. Ethical review for this study was completed by Advarra CIRBI Platform, using the Department of Health and Human Services regulations found at 45 CFR 46.104(d)(2); the IRB determined that the research project is exempt from IRB oversight.

Health Care Providers (*n* = 238)		
Average Number of Patients per Month	330	
	Primary Care Providers	OB-GYNs
Specialty	*n* = 159	*n* = 79
		Respondents
Geographic Region	West	22%
	Midwest	23%
	South	32%
	Northeast	23%
Practice Type	Private Practice	43%
	Academic Health System	32%
	Community Health System	25%
Payers (*n* = 40)		
		Respondents
Geographic Region	West	25%
	Midwest	25%
	South	18%
	Northeast	32%
Geographic Reach	Single US State	23%
	Regional (Multiple States)	20%
	National Organization	57%
Average Plan Size (Lives)	10,000–100,000	8%
	100,001–1,000,000	18%
	1,000,001–5,000,000	32%
	5,000,001–10,000,000	18%
	>10,000,000	24%
Plan Breakdown of Lives Covered	Medicaid	26%
	Commercial	26%
	Medicare	48%
Patients (*n* = 116)		
		Respondents
Sex	Male	38%
	Female	62%
Age	30–39 years old	34%
	40–49 years old	21%
	50–54 years old	9%
	55–64 years old	16%
	65–74 years old	20%
Care Setting	Doctors Office Not at Hospital	53%
	Doctors Office at Hospital	24%
	Public Health Clinic	8%
	Retail Clinic	2%
	Concierge Service	2%
	No Routine Care	11%
Geographic Region	West	20%
	Midwest	33%
	South	33%
	Northeast	14%
Ethnicity	White	66%
	Black/African American	14%
	Asian	7%
	Latin American/Hispanic	6%
	Other	7%
Insurance Type	Private Insurance	37%
	Medicare	22%
	Medicaid	16%
	Medicare and Medicaid	16%
	Uninsured/Self-Insured	7%
	Veteran’s Affairs	2%
Annual Household Income	<$25,000	28%
	$25,001–75,000	38%
	$75,001–125,000	21%
	$125,001–175,000	10%
	>$175,000	3%

## Data Availability

The data underlying this article will be shared on reasonable request to the corresponding author.

## Supplementary Materials

The following supporting information can be downloaded at: https://www.mdpi.com/article/10.3390/jpm14060593/s1, Figure S1: Patient Compliance with HCP Recommended Cancer Screening Tests, Figure S2: Factors Contributing to Patient Compliance with Receiving Any HCP Recommended Cancer Screening Test, Figure S3: Patient-Reported Difficulty to Complete/Improvements to be Made to Any HCP Recommended Cancer Screening Test, Figure S4: HCP and Payer-Reported View of Interest in Cancer Screening Tests for Indications without Guideline Recommendations Today, Figure S5: Influence on HCPs Decision to Incorporate New Cancer Screening Tests prior to USPSTF Guideline Recommendation, Figure S6: HCPs Adoption of New Cancer Screening Tests Prior to USPSTF Guideline Inclusion Important Factors and Most Influential Clinical Utility Data for Blood-Based MCED Test Adoption, Figure S7: Payer Viewpoint on Cost of Cancer Care Management and Factors to Reduce Cost, Figure S8: HCP and Payer Awareness, Adoption, Evaluation, and Coverage of Blood-Based MCED Tests, Figure S9: Patient Excitement and Likelihood to Seek Out Blood-Based MCED Tests, Figure S10: Factors Influence on Patients’ Decision to Receive a Blood-based MCEDS Test, Figure S11: Patient-Ranked HCP Support to Influence for MCED Test Adoption.
