# Supplementary material for: Perspectives on Clinical Adoption Barriers to Blood-Based Multi-Cancer Early Detection Tests across Stakeholders
Source: J Pers Med. 2024 Jun 1;14(6):593. doi: 10.3390/jpm14060593 (PMC11204763; doi:10.3390/jpm14060593)
Supplement: Supplementary file 1 [file jpm-14-00593-s001.zip › Supplementary Files Schroll et al 2024Apr5.pdf]

Supplementary Results:

Figure S1

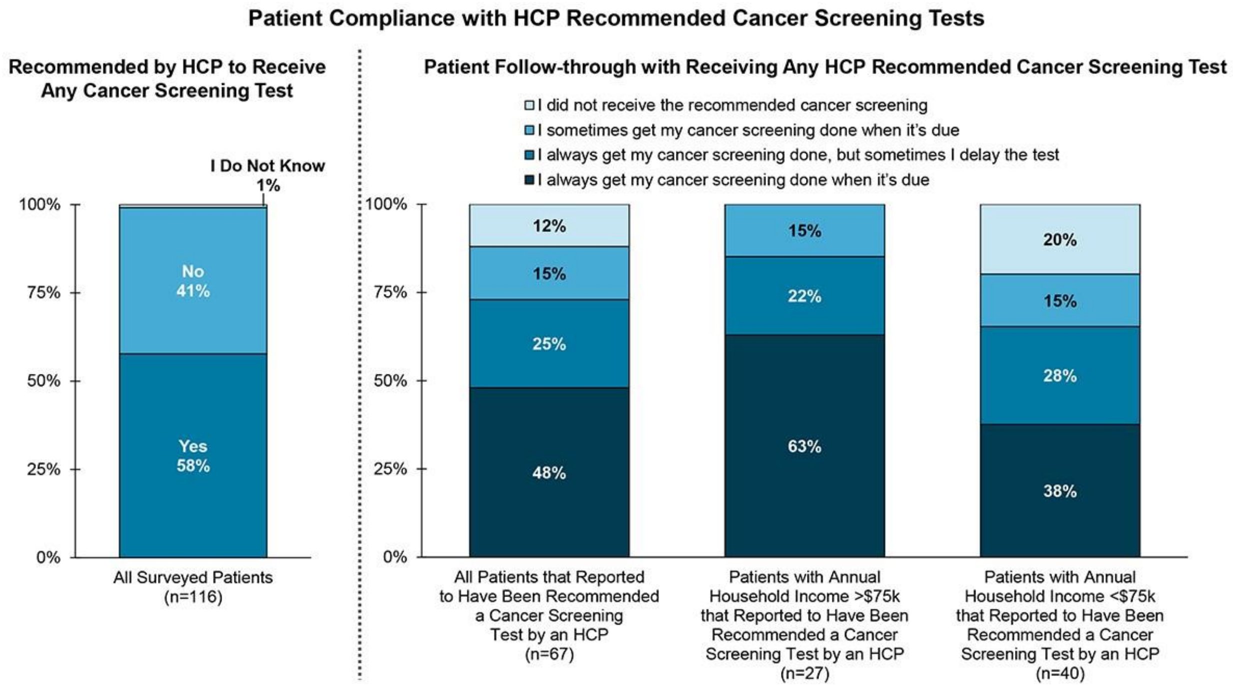

Figure S1: Patient Compliance with HCP Recommended Cancer Screening Tests. 58% of survey patients reported to have ever been recommended to receive a cancer screening test from a HCP (leftmost bar graph). Of those, 12% reported to not follow through with receiving the HCP recommended cancer screening test. Assessing the responses by annual household income (rightmost 2 bar graphs) showed that all patients with an annual income >$75k received the HCP recommended cancer screening testing at some point in time and 20% of patients with an annual income <$75k never received the testing.

Figure S2

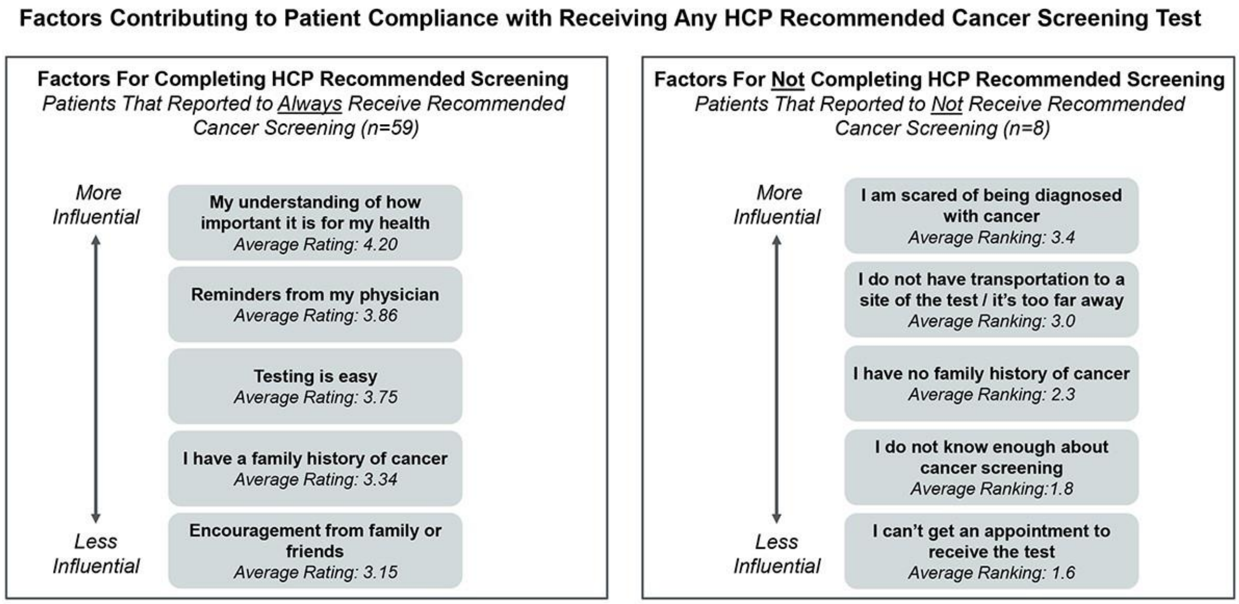

Figure S2: Factors Contributing to Patient Compliance with Receiving Any HCP Recommended Cancer Screening Test. Patients that reported to always receive the HCP recommended cancer screening testing rated 'my understanding of how important it is for my health' as the top influential factor (left). Patients that reported to never complete HCP recommended cancer screening and testing ranked 'I am scared of being diagnosed with cancer' as the top factor (right).

Figure S3

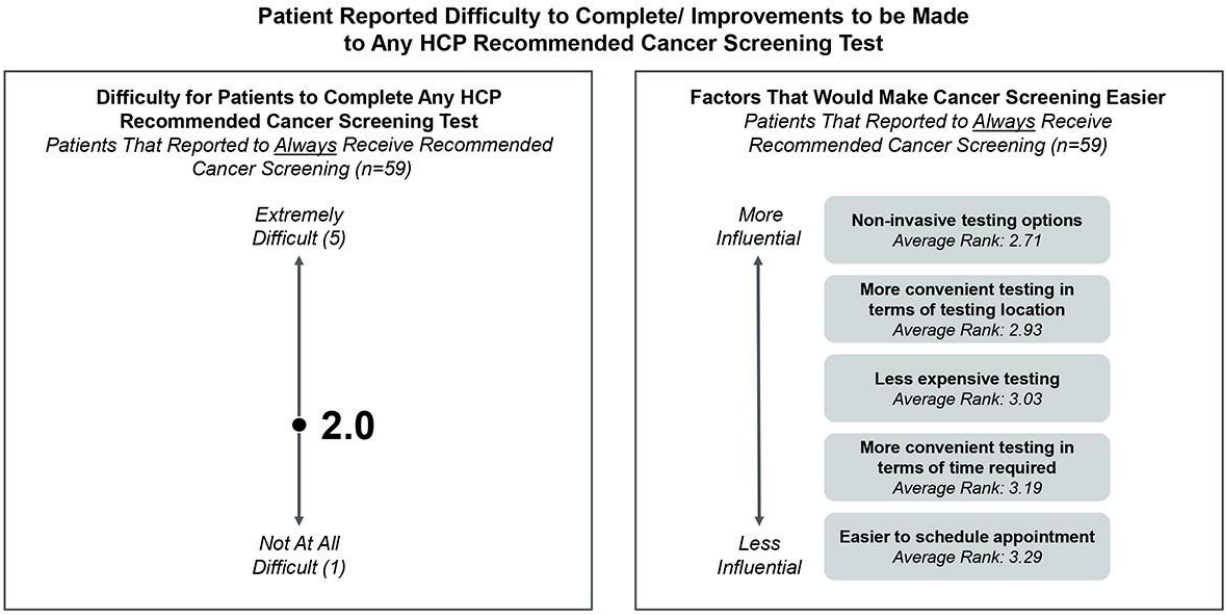

Figure S3: Patient Reported Difficulty to Complete/Improvements to be Made to Any HCP Recommended Cancer Screening Test. Patients that reported to always receive the HCP recommended cancer screening testing rated the difficulty of receiving the test a 2.0/5 (left) and ranked non-invasive testing options and more convenient testing in terms of testing location as the top factors that would make receiving cancer screening testing easier (right).

Figure S4

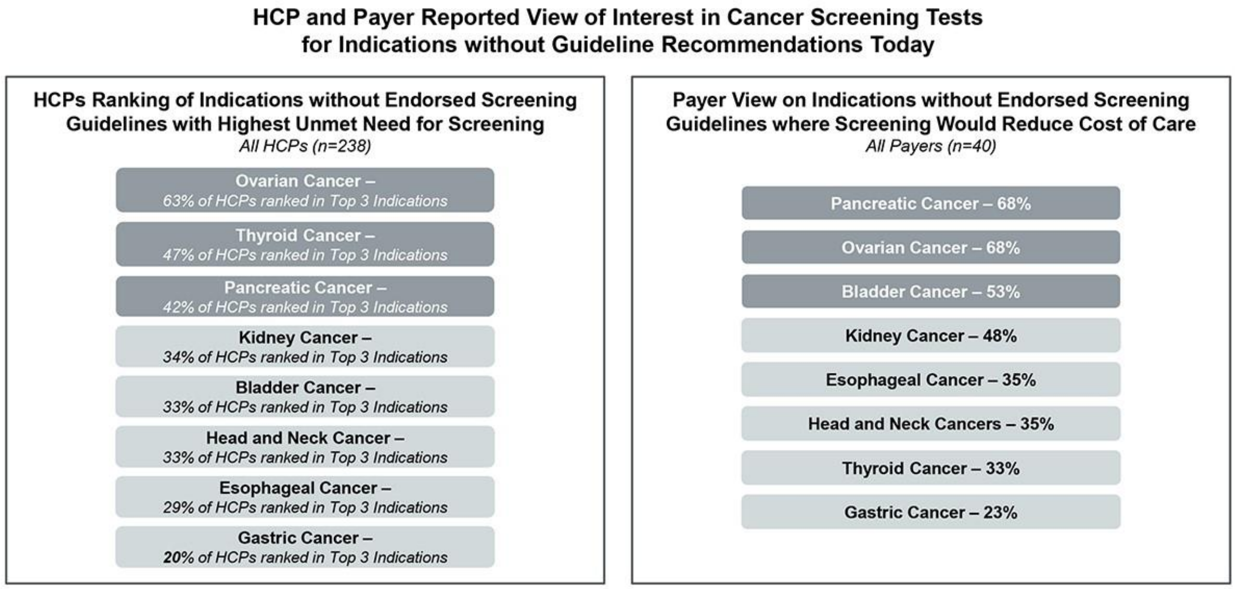

Figure S4: HCP and Payer Reported View of Interest in Cancer Screening Tests for Indications without Guideline Recommendations Today. HCPs ranked ovarian cancer, thyroid cancer, and pancreatic cancer as the highest unmet need indications without endorsed screening guidelines today (left). Similarly, payers were asked to select all indications without endorsed screening today, where screening would reduce the cost of care and pancreatic cancer, ovarian cancer, and bladder cancer were selected as the top three indications (right).

Figure S5

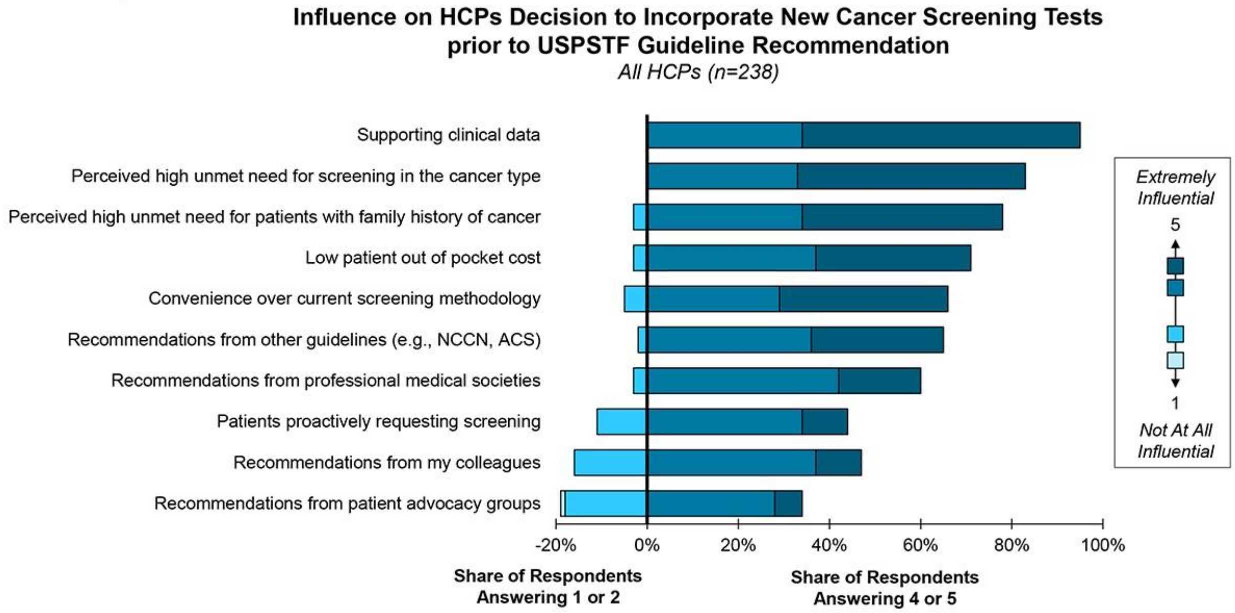

Figure S5: Influence on HCPs Decision to Incorporate New Cancer Screening Tests prior to USPSTF Guideline Recommendation. Surveyed HCPs rated 'supporting clinical data', 'perceived high unmet for screening in the cancer type' and 'perceived high unmet need for patients with family history of cancer' as the most influential factors and 'recommendations from patient advocacy groups' or 'recommendations from my colleagues' as the least influential factors when deciding to incorporate a cancer screening test prior to USPSTF guideline inclusion into their practice. Note: NCCN = National Comprehensive Cancer Network, ACS = American Cancer Society.

Figure S6

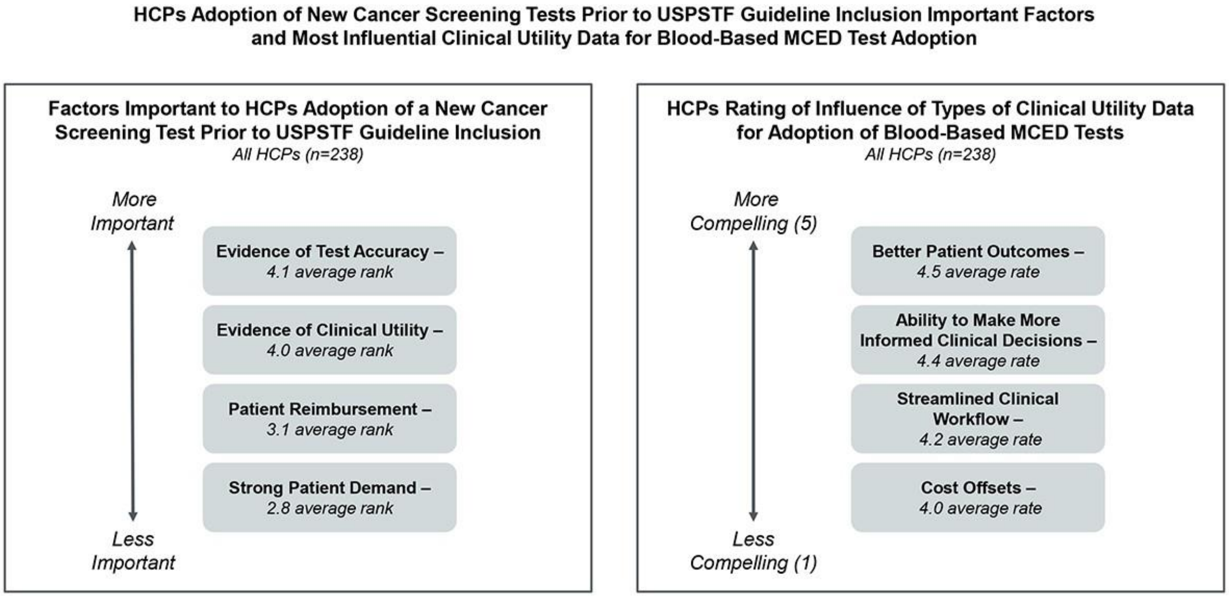

Figure S6: HCPs Adoption of New Cancer Screening Tests Prior to USPSTF Guideline Inclusion Important Factors and Most Influential Clinical Utility Data for Blood-Based MCED Test Adoption. HCPs reported that evidence of test accuracy and clinical utility are the most important factors when considering the adoption of any new cancer screening and early detection test prior to USPSTF guideline recommendations (left). Regarding blood-based MCED tests specifically, HCPs rated better patient outcomes as the most compelling type of clinical utility data for the adoption prior to USPSTF guideline recommendations (right).

Figure S7

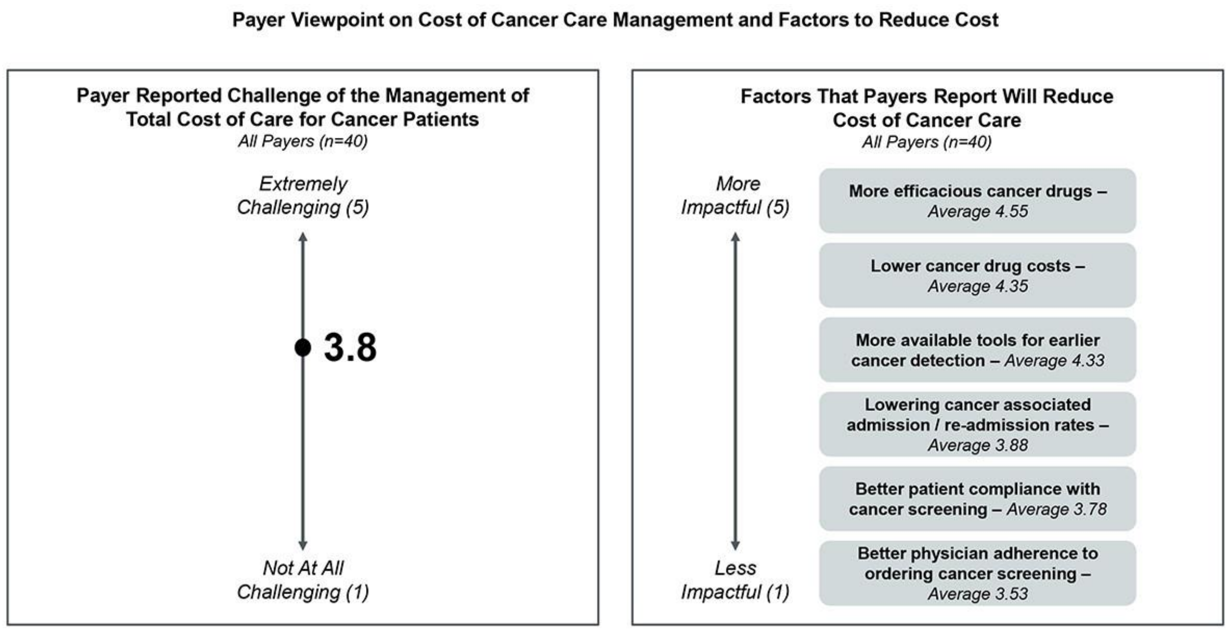

Figure S7: Payer Viewpoint on Cost of Cancer Care Management and Factors to Reduce Cost. Surveyed payers rated the challenge of managing to the total cost of care for cancer patients for their respective plans as a 3.8/5 (left). Payers rated 'more efficacious cancer drugs', 'lower cancer drug costs', and 'more available tools for earlier cancer detection' as the top three factors that would be most impactful for reducing the cost of cancer care for insurance plans (right).

Figure S8

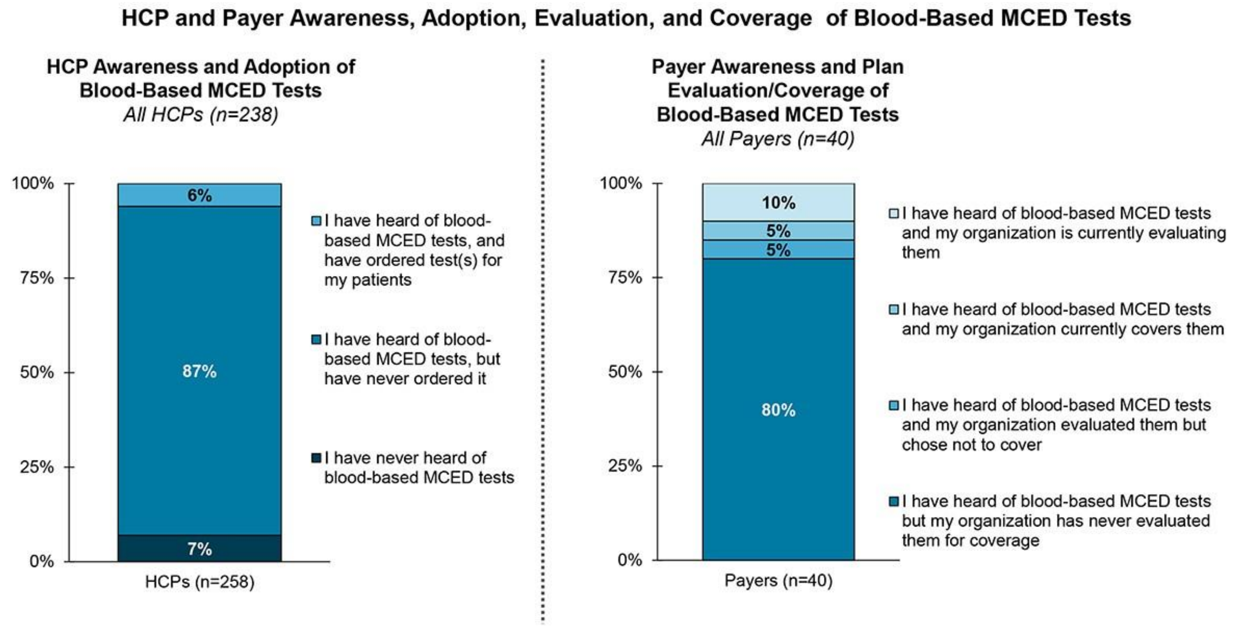

Figure S8: HCP and Payer Awareness, Adoption, Evaluation, and Coverage of Blood-Based MCED Tests. Surveyed HCPs were asked their awareness and current adoption of blood-based MCED tests, 93% reported to be aware of blood-based MCED test and 6% reported to have ordered a test for patients (left). All surveyed payers reported to have heard of blood-based MCED tests, 80% reported that their plans had not yet evaluated them for coverage at the time of the survey (right).

Figure S9

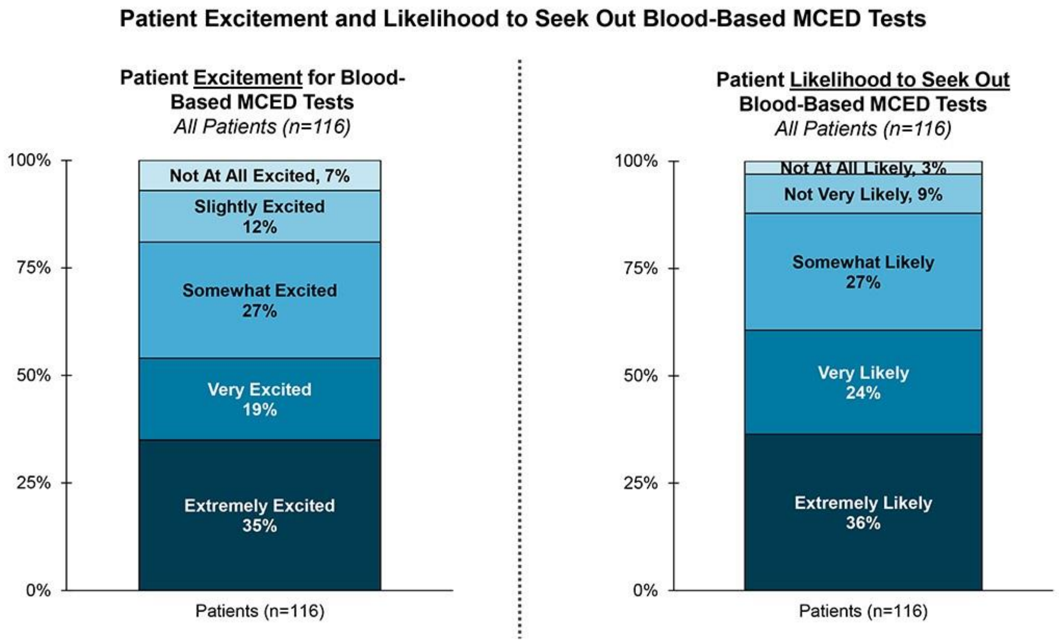

Figure S9: Patient Excitement and Likelihood to Seek Out Blood-Based MCED Tests. The majority of surveyed patients reported some level of excitement for blood-based MCED tests with 7% reporting 'not at all excited' (left). When asked if they would be likely to seek out a blood-based MCED tests majority of patients reported some level of likelihood with 3% reporting 'not at all likely' (right).

Figure S10

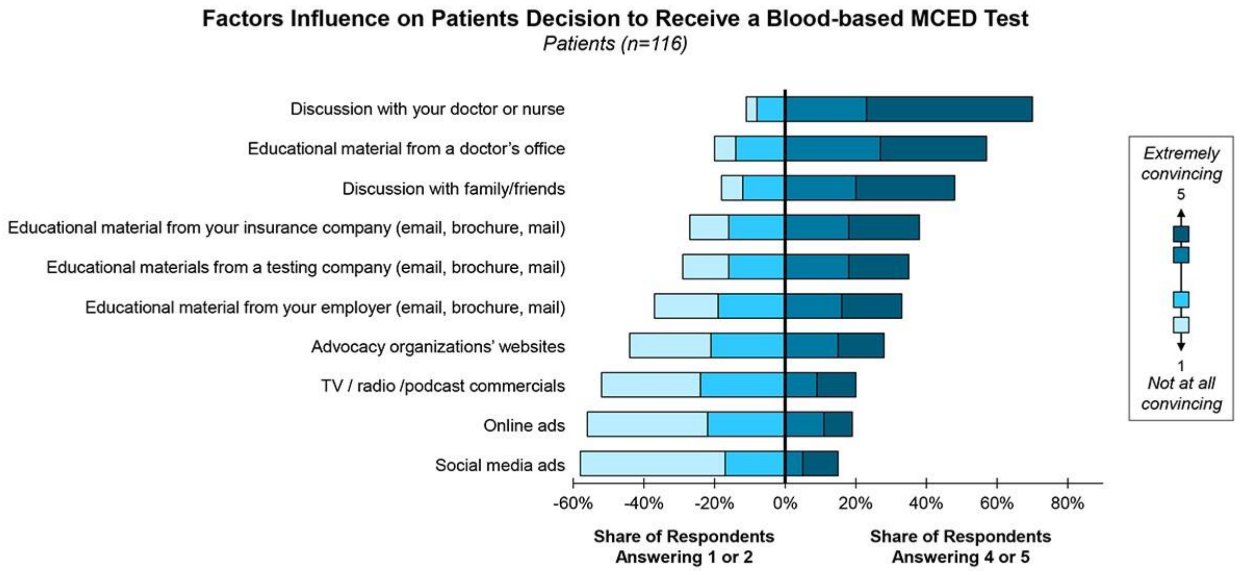

Figure S10: Factors Influence on Patients Decision to Receive a Blood-based MCEDS Test. Surveyed patents rated 'discussion with your doctor or nurse' and 'educational material from a doctor's office' as the most convincing factors and 'social media ads' and 'online ads' as the least convincing factors when deciding to receive a blood-based MCED test.

Figure S11

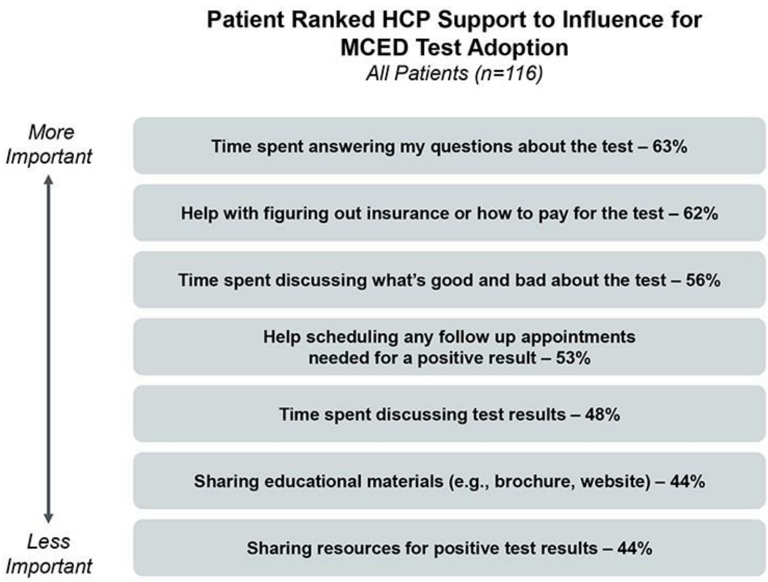

Figure S11: Patient Ranked HCP Support to Influence for MCED Test Adoption. Surveyed patient ranked 'time spent with answering my questions about the test' and 'help figuring out insurance or how to pay for the test' as the top most important type of support that HCPs could offer to impact their willingness to receive a blood-based MCED test.

Supplementary Methods: Healthcare Provider, Payer, and Patient Survey Questionnaires

S1: Healthcare Provider Survey Questionnaire

1. Which of the following best describes your primary medical specialty? *Please select only one.*
   - **a.** Adult Primary Care / Internal Medicine
   - b. Family Medicine
   - c. Pediatric Primary Care **[Terminate from Survey if selected]**
   - d. Ob/Gyn
   - **e.** Pulmonologist **[Terminate from Survey if selected]**
   - f. Family Medicine Nurse Practitioner (NP)
   - g. Family Medicine Physician Assistant (PA)
   - **h.** Other (please specify): **[Terminate from Survey if selected]**
2. Are you board certified to practice in your primary medical specialty?
   - a. Yes
   - b. No **[Terminate from Survey if selected]**
3. How many years have you been in practice post-fellowship (if applicable)?
   - a. 0-2 years **[Terminate from Survey if selected]**
   - b. 3-10 years
   - c. 11-20 years
   - d. 21-35 years
   - e. More than 35 years **[Terminate from Survey if selected]**
4. What percent of your professional time is spent dedicated to direct patient care?
   - a. Less than 25% **[Terminate from Survey if selected]**
   - b. 25-50% **[Terminate from Survey if selected]**
   - c. 50-75%
   - d. More than 75%
5. In what region is your practice located?
   - a. Northeast (CT, MA, ME, NH, NJ, NY, PA, RI, VT)
   - b. South (AL, AR, DC, DE, FL, GA, KY, LA, MD, MI, NC, OK, SC, TN, TX, VA, WV)
   - c. Midwest (IA, IL, IN, KS, MI, MN, MO, NE, ND, OH, SD, WI)
   - d. West (AK, AZ, CA, CO, HI, ID, MT, NM, NV, OR, UT, WA, WY)
   - e. Outside the US **[Terminate from Survey if selected]**
6. In which state is your practice located?
   - a. CT
   - b. MA
   - c. ME **[Terminate from Survey if selected]**
   - d. NH
   - e. NJ
   - f. NY
   - g. PA
   - h. RI
   - i. VT **[Terminate from Survey if selected]**
7. Which best describes your primary practice setting? *Please select only one.*
   - a. Academic Hospital
   - b. Community Hospital
   - c. Private Practice, Hospital- or Network-Owned / Affiliated
   - d. Independent Private Practice
   - e. Other (please specify) **[Terminate from Survey if selected]**

8. How many patients do you see in an average month?

[NUMERICAL] patients/month [Terminate from <30 patients/months]

9. For which of the following cancers do you routinely offer screening (checking for cancer or abnormal cells that may become cancer in people who have no symptoms)? Please select all that apply.
    a. Colorectal Cancer
    b. Breast Cancer
    c. Ovarian Cancer
    d. Lung Cancer
    e. Pancreatic Cancer
    f. Cervical Cancer
    g. Thyroid Cancer
    h. Prostate Cancer
    i. Other (please specify)

10. Which of the following cancer early detection and screening tests do you currently offer today? *Please select all that apply.*

    a. 2D Mammography
    b. 3D Mammography
    c. Low-dose CT
    d. Colonoscopy
    e. FIT / FOBT
    f. Cologuard
    g. Cervical Cytology
    h. Cancer Antigen 125 (CA-125)
    i. Shield (Blood-based Liquid Biopsy for Colorectal Cancer)
    j. IMMray PanCan-d (Blood-based Liquid Biopsy for Pancreatic Cancer)
    k. Oncoguard Liver (Blood-based Liquid Biopsy for Liver Cancer)
    l. Galleri (Multi-cancer early detection test)
    m. Other (please specify)
    n. None of the above **[Terminate from Survey if selected]**

Congratulations! You have qualified for our survey. This survey will take approximately 20 minutes to complete. You must complete the entire survey to qualify for the honorarium.

We are conducting this survey to support development of a publication to generate awareness about cancer screening and early detection tests and engage relevant stakeholders. In order to successfully carry out this mission, we must have a deep understanding of current trends in cancer screening and early detection implementation, including perceived value and barriers to adoption.

This survey will evaluate cancer screening and early detection adoption and perception. We implore respondents to accurately represent the health systems at which they are employed. We understand that you may wish to confer with others in your health system. You will be able to leave and return to this link to complete the survey at any time.

The possible risks of the study are minimal. Furthermore, your participation is strictly voluntary and you may withdraw your participation at any time without penalty.

The results of this survey will only be shared on an aggregated, anonymous basis. Your responses will be treated as confidential and will not be shared in any format that could be directly linked back to you or your practice.

By clicking NEXT you are verifying that you have read the explanation of the study, and that you agree to participate. You also understand that your participation in this study is strictly voluntary.

Thank you for your participation.

For this survey we are interested in cancer screening and early detection defined as: The identification of a cancer-associated signal that requires diagnostic testing for confirmation of the presence and type of cancer, at an early stage for that specific cancer type in an individual with no signs or symptoms of cancer at the time the test is performed, regardless of risk. Examples of single cancer detection tests include mammography for breast cancer, low-dose CT for lung cancer, cervical cytology for cervical cancer, and colonoscopy/Cologuard/FIT for colorectal cancer.

1. For the cancers you are regularly screening for today, please rate the following challenges you face.

	**1 Not challenging**	**2**	**3 Somewhat challenging**	**4**	**5 Extremely challenging**
Identifying the appropriate patients for screening					
Keeping track of screening frequency due to patient characteristics (e.g. higher frequency due to family history)					
Time spent educating patient about test					
Ensuring patient compliance					
Reimbursement					
Test performance (sensitivity / specificity)					
Time spent delivering results to patient					
Managing patient psychological burden after a positive / abnormal result					
Managing follow-up tests after a positive / abnormal result					
Referring patients to specialists in a timely fashion after a positive / abnormal result					
Other (please specify)					

2. How influential would each of the following be on your decision to incorporate a cancer screening test into routine practice prior to USPSTF guideline inclusion?

	1<br>**Not at all influential**	**2**	**3**<br>**Somewhat influential**	**4**	**5**<br>**Extremely influential**
High unmet need for screening in the cancer type					
a) Supporting clinical data					
b) Convenience over current screening methodology					
c) Proactive patients request screening					
d) Patients have a family history of cancer					
e) Recommendations from other guidelines *(e.g., NCCN, ASC)*					
f) Recommendations from my colleagues					
g) Recommendations from professional medical societies					
h) Recommendations from patient advocacy groups					
i) Other (please specify)					

3. How influential would each of the following be on your decision to incorporate a cancer screening test into routine practice prior to USPSTF guideline inclusion? *Please drag each from the left to the right, starting with the most important on top.*
    a. High unmet need for screening in the cancer type
    b. Supporting clinical data
    c. More convenient test for my patients than current screening methodology
    d. Patients have a family history of cancer
    e. Recommendations from other guidelines *(e.g., NCCN, ASC)*
    f. Recommendations from my colleagues
    g. Recommendations from professional medical societies
    h. Recommendations from patient advocacy groups
    i. Other (please specify)

4. Which of the following concerns would prevent you from incorporating a cancer screening testing into your clinical practice prior to USPSTF guideline inclusion? Please drag each from the left to the right, starting with the most important on top.
    a. Lack of clinical evidence
    b. Lack of clinical utility
    c. Lack of guidelines
    d. Lack of patient demand
    e. Lack of reimbursement

f. Other (please specify)

5. Which of the following cancers, where screening is not currently endorsed by guidelines, are you most interested in screening for? *Please drag each from the left to the right, starting with the most important on top.*
    a. Bladder cancer
    b. Kidney cancer
    c. Thyroid cancer
    d. Pancreatic cancer
    e. Ovarian cancer
    f. Esophageal cancer
    g. Stomach cancer
    h. Head and neck cancers
    i. Other (please specify)

The following questions are in regard to **liquid biopsy multi-cancer early detection tests** defined as: a diagnostic test that purports to screen and detect simultaneously for two or more cancer types by identifying cancer signals found in blood. Blood-based multi-cancer early detection and screening tests are complementary to existing cancer screenings and designed to detect many tumor types including cancers that are not commonly screened for today.

6. Which of the following best describes your awareness of blood-based multi-cancer early detection tests?
    a. I have never heard of blood-based multi-cancer early detection tests
    b. I have heard of blood-based multi-cancer early detection tests, but have never ordered it
    c. I have heard of blood-based multi-cancer early detection tests, and have ordered test(s) for my patients

7. **[If above question = C]** How many blood-based multi-cancer early detection tests have you ordered in the past year?

[NUMERICAL] tests in the past year

8. Please rate how much you agree with the following statement.

	**1 Strongly disagree**	**2**	**3 Neither agree nor disagree**	**4**	**5 Strongly agree**
Using a blood-based multi-cancer early detection test would allow me to detect numerous cancers earlier, which would improve patient outcomes with earlier intervention					

9. Please rank which patient segments are most appropriate for a blood-based multi-cancer early detection test for? *Please drag each from the left to the right, starting with the most important on top.*
    a. Patients with an identified mutation in a hereditary cancer gene (e.g. BRCA)
    b. Patients with a family history of cancer

c. Patients with unexplained symptoms *(e.g., weight loss, change in appetite)*
d. Patients who are 30 years or older
e. Patients who are 40 years or older
f. Patients who are 50 years or older
g. Patients who are 65 years or older
h. Other (please specify)

10. Please rate how compelling each of the following types of clinical utility data are in the context of a blood-based multi-cancer early detection test.

	**1 Not at all compelling**	**2**	**3 Somewhat compelling**	**4**	**5 Extremely compelling**
Improved efficacy in clinical decision making					
Streamlined clinical workflow (e.g. accelerating time to cancer diagnosis)					
Better patient outcomes					
Cost offsets					

11. Assume you are satisfied with the available data and you intend to incorporate blood-based multi-cancer early detection tests into routine clinical practice in the future, please rate how concerning each of the following will be.

	**1 Not at all concerning**	**2**	**3 Somewhat concerning**	**4**	**5 Extremely concerning**
Ease of ordering testing					
Lack of reimbursement / high cost to patient					
Utilization management hurdles *(e.g., prior authorization)*					
Lack of clarity on patient eligibility					
Lack of clarity on test frequency					
Inability to act on positive test results					
Time required to educate patients about test					
Patient hesitation to be screened for multiple cancers					
Patient concerns about false positive and/or false negative test results					
Patient psychological burden after receiving a positive result					
Referring patient to appropriate specialist					
Being responsible for follow-up testing after a positive / abnormal result					
Other (please specify)					

12. Please rank your top 5 concerns when considering incorporating blood-based multi-cancer early detection tests into routine clinical practice in the future. *Please drag each concern from the left to the right, starting with the top concern.*
    a. Ease of ordering testing
    b. Lack of reimbursement / High cost to patient
    c. Utilization management hurdles (e.g.; prior authorization)
    d. Lack of guidelines on patient eligibility
    e. Lack of guidelines on test frequency
    f. Inability to act on results
    g. Time required to educate patients about test
    h. Patient hesitation to be screened for multiple cancers

i. Patient concerns about false positive and/or false negative test results
j. Patient psychological burden based on positive result
k. Referring to appropriate specialist
l. Being responsible for follow-up testing based on positive result
m. Other (please specify)

S2: Payer Survey Questionnaire

1. Which of the following best describes the payer organization you are currently employed at?
   a) Commercial Payer
   b) Integrated Delivery Network (IDN)
   c) Medicare Administrative Contractor (MAC)
   d) Medicaid / Managed Care Organization (MC)
   e) Other (please specify)

2. Which of the following best describes your primary role at your organization? *Please select only one.*
   a) Medical Director
   b) Claims Manager **[Terminate from Survey if selected]**
   c) Clinical Advisor
   d) Health Economics Outcomes Research (HEOR) and Market Access Manager **[Terminate from Survey if selected]**
   e) Chief Medical Officer
   f) VP of Clinical Services **[Terminate from Survey if selected]**
   g) Laboratory Benefits Manager
   h) Pharmacy Director **[Terminate from Survey if selected]**
   i) Other **[Terminate from Survey if selected]**

3. How many years of experience do you have working at a payer organization, including your current organization and ones you may have worked at in the past?
   a. <2 years **[Terminate from Survey if selected]**
   b. 2-5 years
   c. 5-10 years
   d. >10 years

4. To what extent are you involved in policy, reimbursement, formulary placement, and coverage decisions for your plan?
   a. I have no role at all in these decisions **[Terminate from Survey if selected]**
   b. I rarely serve in an advisory capacity **[Terminate from Survey if selected]**
   c. I frequently serve in an advisory capacity
   d. I am directly involved in decision making some or all of the time

5. Please select the products for which you are involved in policy, reimbursement, and coverage decisions. *Please select all that apply.*
   a. Biologic drugs
   b. Small molecule drugs
   c. Medical devices
   d. Diagnostic tests **[Terminate from Survey if not selected]**
   e. I am not involved in medical policy for products, only procedures **[Terminate from Survey if only selected]**

6. Which of the following areas of diagnostic tests do you oversee? *Please select all that apply.*
    a. Oncology **[Terminate from Survey if not selected]**
    b. Infectious disease
    c. Autoimmune
    d. Diagnostic imaging
    e. Genetic testing
    f. Clinical laboratory tests
    g. Other (please specify):

7. How many lives are covered by the plan or organization for which you are involved in policy decisions?
    a. <10,000 lives **[Terminate from Survey if selected]**
    b. 10,000-100,000 lives
    c. 100,000-1,000,000 lives
    d. 1,000,000-5,000,000 lives
    e. 5,000,000-10,000,000 lives
    f. >10,000,000 lives

8. What percentage of your plan's covered lives are represented by each? Column must total 100%.
    a. Medicare
    b. Medicaid
    c. Commercial
    d. Other (please specify)

9. What is the geographic reach of your organization?
    a. Single US State
    b. Regional (multiple concentrated states)
    c. National (multiple states across the country)
    d. Outside the US [Terminate from Survey if selected]

10. In what region of the United States do you live?
    b. Northeast (CT, MA, ME, NH, NJ, NY, PA, RI, VT)
    c. South (AL, AR, DC, DE, FL, GA, KY, LA, MD, MI, NC, OK, SC, TN, TX, VA, WV)
    d. Midwest (IA, IL, IN, KS, MI, MN, MO, NE, ND, OH, SD, WI)
    e. West (AK, AZ, CA, CO, HI, ID, MT, NM, NV, OR, UT, WA, WY)

Congratulations! You have qualified for our survey. This survey will take approximately 20 minutes to complete. You must complete the entire survey to qualify for the honorarium.

We are conducting this survey to support development of a publication to generate awareness about cancer screening and early detection tests and engage relevant stakeholders. In order to successfully carry out this mission, we must have a deep understanding of current trends in cancer screening and early detection tests implementation, including perceived value and barriers to adoption.

This survey will evaluate cancer screening and early detection tests adoption and perception. We implore respondents to accurately represent the payer systems at which they are employed. We understand that you may wish to confer with others in your health system. You will be able to leave and return to this link to complete the survey at any time.

The possible risks of the study are minimal. Furthermore, your participation is strictly voluntary and you may withdraw your participation at any time without penalty.

The results of this survey will only be shared on an aggregated, anonymous basis. Your responses will be treated as confidential and will not be shared in any format that could be directly linked back to you or your practice.

By clicking NEXT you are verifying that you have read the explanation of the study, and that you agree to participate. You also understand that your participation in this study is strictly voluntary.

Thank you for your participation.

1. How challenging is it for your plan to manage the total cost of care for cancer patients, on a scale of 1 to 5, where 1 is not significant and 5 is very significant.

	**1 – Not at all Challenging**		**3 – Somewhat challenging**		**5 – Very challenging**
Cancer Total Cost of Care					

2. What percentage of your plan's overall medical spend *(e.g., screenings, diagnostics, procedures, etc)* is represented by cancer care? **[0-100%]**

3. Please rate the following factors in terms of their potential impact on reducing the <u>cost burden of cancer care for your health plan</u>, where 1 is no impact at all and 5 is very impactful.

	**1 – No Impact At All**		**3 – Somewhat Impactful**		**5 – Very Impactful**
More available tests for earlier cancer detection					
Better patient compliance with cancer screening					
Better physician adherence to ordering cancer screening					
More efficacious cancer drugs					
Lower cancer drug costs					
Lowering cancer associated admission/ re-admission rates					
Other (please specify)					

4. Does your organization cover any of the following cancer screening and early detection tests that are not USPSTF grade A/B? *Please select all that apply.*

a) 3D Mammography for Breast Cancer
b) Cancer Antigen 125 (CA-125) for Ovarian Cancer
c) Shield (Blood-based Liquid Biopsy for Colorectal Cancer)
d) IMMray PanCan-d (Blood-based Liquid Biopsy for Pancreatic Cancer)
e) Oncoguard Liver (Blood-based Liquid Biopsy for Liver Cancer)
f) Galleri (Blood-based multi-cancer early detection test)
g) Other (please specify)
h) None of the above

5. *The following questions are in regard to* ***liquid biopsy multi-cancer early detection and screening tests*** *defined as: a diagnostic test that purports to screen and detect simultaneously for two or more cancer types by identifying cancer signals found in blood. Blood-based multi-cancer early detection and screening tests are complementary to existing cancer screenings and designed to detect many tumor types including cancers that are not commonly screened for today.*

6. Which of the following best describes your awareness of blood-based multi-cancer early detection and screening tests?
   a) I have never heard of blood-based multi-cancer early detection and screening tests
   b) I have heard of blood-based multi-cancer early detection and screening tests but my organization has never evaluated them for coverage
   c) I have heard of blood-based multi-cancer early detection and screening tests and my organization evaluated them but chose not to cover
   d) I have heard of blood-based multi-cancer early detection and screening tests and my organization currently covers them

7. Please rate how much you agree with the following statement, on a scale from 1 to 5, where 1 is strongly disagree and 5 is strongly agree.

	**1 Strongly disagree**	**2**	**3 Neither agree nor disagree**	**4**	**5 Strongly agree**
Using a blood-based multi-cancer early detection and screening test will allow the detection of numerous cancers earlier, which will lead to long-term cost savings by improving patient outcomes with earlier intervention					

8. For which of the following cancer types not currently included in USPSTF screening guidelines would a test for earlier detection help to reduce cost of care? *Please select all that apply.*
   Bladder cancer
   a. Kidney cancer
   b. Thyroid cancer
   c. Pancreatic cancer
   d. Ovarian cancer
   e. Esophageal cancer

f. Stomach cancer
g. Head and neck cancers
h. Other (please specify)

9. For which patient segments would you be willing to cover a blood-based multi-cancer early detection and screening test? *Please select all that apply*.
    a. Patients with a genetic predisposition to a single cancer
    b. Patients with a genetic predisposition to multiple cancers
    c. Patients with a family history of a single cancer
    d. Patients with a family history of multiple cancers
    e. Patients with unexplained symptoms (e.g., weight loss, change in appetite)
    f. Patients who are 30 years or older
    g. Patients who are 40 years or older
    h. Patients who are 50 years or older
    i. Patients who are 65 years or older
    j. Patients at high risk for a cancer type not currently covered by USPSTF screening guidelines
    k. Other (please specify)
    l. None of the above

10. Please rate what you would need to see happen in order for you to be comfortable covering multi-cancer detection tests prior to USPSTF guideline inclusion, on a scale from 1 to 5, where 1 is not at all important and 5 is extremely important.

	1<br>Not at all important	2	3<br>Somewhat important	4	5<br>Extremely important
Robust clinical evidence demonstrating efficacy					
Robust clinical evidence demonstrating clinical utility					
Evidence of long-term cost savings					
FDA approval and CMS review					
Clear patient segmentation from test manufacturer					
Clear testing frequency guidelines from test manufacturer					
Clear referral pathways to specialists after a positive result					
Low rate of false positives					
Low rate of false negatives					
Physician demand					
Low cost of test					
Other (please specify)					

11. Please rank, with the most important on top, in terms of what you would need to see happen in order for you to be comfortable covering multi-cancer early detection and screening tests prior to USPSTF guideline inclusion. *Please drag each phrase from the left to the right, starting with the most important.*
    a. Robust clinical evidence demonstrating efficacy
    b. Robust clinical evidence demonstrating clinical utility
    c. Evidence of long-term cost savings
    d. FDA approval and CMS review
    e. Clear patient segmentation from test manufacturer
    f. Clear testing frequency guidelines from test manufacturer
    g. Clear referral pathways to specialists after a positive result
    h. Low rate of false positive
    i. Low rate of false negatives
    j. Physician demand
    k. Low cost of test
    l. Other (please specify) **S3: Patient Survey Questionnaire**

12. What is your age?
    a. 0-14 years old **[Terminate from Survey if selected]**
    b. 15-24 years old **[Terminate from Survey if selected]**
    c. 25-34 years old **[Terminate from Survey if selected]**
    d. 30-39 years old
    e. 40-44 years old
    f. 45-49 years old
    g. 50-54 years old

h. 55-64 years old
i. 65-74 years old
j. 75-84 years old **[Terminate from Survey if selected]**
k. 85 years or older **[Terminate from Survey if selected]**

13. With which gender do you most closely identify?
    a. Male
    b. Female
    c. Transgender male
    d. Transgender female
    e. Gender variant/non-conforming
    f. Other (please specify)
    g. Prefer not to answer

14. With which racial or ethnic group do you most closely identify?
    a. American Indian or Alaska Native
    b. Asian
    c. Black or African American
    d. Hispanic or Latino
    e. Middle East or North Africa
    f. Native Hawaiian or Other Pacific Islander
    g. White

15. In what region of the United States do you live?
    a. Northeast (CT, MA, ME, NH, NJ, NY, PA, RI, VT)
    b. South (AL, AR, DC, DE, FL, GA, KY, LA, MD, MI, NC, OK, SC, TN, TX, VA, WV)
    c. Midwest (IA, IL, IN, KS, MI, MN, MO, NE, ND, OH, SD, WI)
    d. West (AK, AZ, CA, CO, HI, ID, MT, NM, NV, OR, UT, WA, WY)
    e. I do not live in the United States **[Terminate from Survey if selected]**

16. How would you describe the area in which you live?
    a. Urban
    b. Suburban
    c. Rural

17. How would you describe your current employment status?
    a. Employed full-time (35 hours a week or more)
    b. Employed part-time (less than 35 hours a week)
    c. Self-employed
    d. Unemployed and looking for work
    e. Unemployed and not currently looking for work
    f. Unpaid caregiver (e.g., stay-at-home mom)
    g. A student
    h. Retired
    i. Unable to work (e.g., disabled)

18. What is your annual household income? Please include the combined income (before taxes) from all members of your household.
    a. <$25,000
    b. $25,001-75,000

    c. $75,001-125,000
    d. $125,001-175,000
    e. >$175,000
    f. Prefer not to say

19. What type of health insurance do you currently have?
    a. Private insurance provided by employer or bought on an exchange (e.g., Blue Cross, UnitedHealthCare)
    b. Medicare and/or Medicare Advantage
    c. Medicare and Medicaid
    d. Medicaid only
    e. Veteran's Administration or Other Government Insurance
    f. Uninsured/Self-insured
    g. Other (please specify)

20. Where do you typically receive your routine (non-urgent) care, such as annual physicals?
    a. Office affiliated with a hospital
    b. Office not affiliated with a hospital
    c. A retail clinic (e.g., CVS Minute Clinic, Walmart Health Center)
    d. A public health clinic (government funded with free or very low-cost care)
    e. Membership or concierge model (e.g., One Medical) in which you pay an annual fee for round-the-clock access to your provider
    f. Other (please specify)
    g. I do not receive routine (non-urgent) care
21. Have you ever been diagnosed with cancer?
    a. Yes **[Terminate from Survey if selected]**
    b. No

22. Please indicate which cancers, if any, your family members have now or have had in the past. *"Family members" are anyone related to you by blood. Please do not include family members who are adopted or are part of your stepfamily.*
    a. Colorectal Cancer
    b. Breast Cancer
    c. Ovarian Cancer
    d. Lung Cancer
    e. Pancreatic Cancer
    f. Cervical Cancer
    g. Thyroid Cancer
    h. Other (please specify)
    i. No family history of cancer

Congratulations! You have qualified for our survey. This survey will take approximately 15 minutes to complete. You must complete the entire survey to qualify for the honorarium.

We are conducting this survey to support development of a publication to generate awareness about cancer screening and early detection tests and engage relevant stakeholders. In order to successfully carry out this mission, we must have a deep understanding of current trends in cancer screening and early detection tests, including perceived value and barriers to adoption.

This survey will evaluate cancer screening and early detection tests adoption and perception. You will be

able to leave and return to this link to complete the survey at any time.

The possible risks of the study are minimal. Furthermore, your participation is strictly voluntary and you may withdraw your participation at any time without penalty.

The results of this survey will only be shared on an aggregated, anonymous basis. Your responses will be treated as confidential and will not be shared in any format that could be directly linked back to you or your practice.

By clicking NEXT you are verifying that you have read the explanation of the study, and that you agree to participate. You also understand that your participation in this study is strictly voluntary.

Thank you for your participation.

1. Has a health care professional recommended you to be screened for a particular cancer in the past?
    a. Yes
    b. No
    c. I do not know
2. **[If above question = Yes]** Which of the following best describes you?
    a. I always get my cancer screening done when it's due
    b. I always get my cancer screening done, but sometimes I delay the test
    c. I sometimes get my cancer screening done when it's due
    d. I did not receive the recommended cancer screening

3. **[If have been recommended for screening]** Which of the following cancers have you ever been screened for in the past?
    e. Colorectal Cancer
    f. Breast Cancer
    g. Ovarian Cancer
    h. Lung Cancer
    i. Pancreatic Cancer
    j. Cervical Cancer (pap smear)
    k. Thyroid Cancer
    l. Prostate Cancer
    m. Other (please specify)
    n. None of the above

4. **[If Q2 = A,B, or C]** What motivates you to complete recommended cancer screening? Please rate the following factors, with 1 being not at all motivating and 5 being extremely motivating.

	**1 Does not motivate me at all**	**2**	**3 Somewhat motivates me**	**4**	**5 Extremely motivates me**
My understanding of how important it is for my health					
Encouragement from family or friends					
Encouragement from a community leader (e.g., pastor)					
Ease of completing testing					
Reminders from my physician that help me complete testing					
Reminders from other sources that help me complete testing					
I have a family history of cancer					

I have a known genetic predisposition to cancer	
Other (please specify)	

5. **[If Q2 = A,B, or C]** How challenging did you find it to complete your cancer screening test? Please rate the following factors, with 1 being not at all challenging and 5 being extremely challenges.

	**1 Not at all Challenging**	**2**	**3 Somewhat Challenging**	**4**	**5 Extremely Challenging**
Completing Clinician Recommended Cancer Screening Test					

6. **[If Q2 = A,B, or C]** Please rank, with the most important at the top, the following improvements you would like to see in cancer screening. Please drag each improvement from the left to the right, starting with the most important improvement.
   a. Easier to schedule appointment
   b. Non-invasive testing options (e.g. blood draw)
   c. Less expensive testing
   d. More convenient testing in terms of time required
   e. More convenient testing in terms of testing location (in-home vs at an office or clinic)
   f. Other (please specify)

7. **[IF Q2 = D]** Please rank **up to 3** of the following reasons for why you did not receive the recommended cancer screening.
   a. I feel healthy and do not think it is necessary
   b. I am scared of getting diagnosed with cancer
   c. I have no family history of cancer
   d. I don't trust the healthcare system
   e. Cancer screening tests are too expensive
   f. I'm too busy for cancer screening tests
   g. I do not have transportation to a site of the test, or it is too far away from my home
   h. I can't get an appointment to receive the test
   i. I do not know enough about cancer screening
   j. I am concerned that getting cancer screening will impact my immigration status
   k. Other (please specify)

The following questions are about blood-based mutli-cancer early detection and screening tests defined as: a blood test that screens for multiple different cancer types at once, early in the disease course, by looking for cancer signals in the blood.

8. How excited are you about blood-based multi-cancer early detection and screening tests?
   a. Extremely excited
   b. Very excited
   c. Somewhat excited

d. Slightly excited
e. Not at all excited

9. Please rate how much you agree with the following statement, on a scale from 1 to 5, where 1 is strongly disagree and 5 is strongly agree. attractive

	**1 Strongly Disagree**	**2**	**3 Neutral**	**4**	**5 Strongly Agree**
Using a blood-based multi-cancer early detection and screening test would allow me to detect numerous cancers earlier, in order to improve cancer outcomes with earlier intervention					

10. How likely would you be to seek out a blood-based multi-cancer early detection and screening?
    a. Not at all likely
    b. Not very likely
    c. Somewhat likely
    d. Very likely
    e. Extremely likely

11. If your provider gave you the option for a blood-based multi-cancer early detection and screening test for your next screening, and it was fully covered by insurance, how likely would you be to choose it?
    a. Extremely likely
    b. Very likely
    c. Somewhat likely
    d. Not very likely
    e. Not at all likely
12. If your provider gave you the option for a blood-based multi-cancer early detection and screening test for your next screening, and you were required to pay <$50 out-of-pocket for the test, how likely would you be to choose it?
    a. Extremely likely
    b. Very likely
    c. Somewhat likely
    d. Not very likely
    e. Not at all likely
13. If your provider gave you the option for a blood-based multi-cancer early detection and screening test for your next screening, and you were required to pay $500 out-of-pocket for the test, how likely would you be to choose it?
    a. Extremely likely
    b. Very likely
    c. Somewhat likely

d. Not very likely
e. Not at all likely

14. Please rate the following concerns that could possibly prevent you from getting blood-based multi-cancer early detection and screening test, with 1 being not at all a concern and 5 being an extreme concern.

	**1 Not at all a concern**	**2 Small concern**	**3 Moderate concern**	**4 Significant concern**	**5 Extreme concern**
Follow-up testing required for positive result					
Fear of needles/blood draws					
Possibility of being diagnosed with life-threatening disease(s)					
Potential for false positive (you don't actually have cancer) and undergoing unnecessary follow up tests					
Potential for false negative (you have cancer, but it was missed)					
Cost of test					
Psychological burden of a positive result					
I don't think I need to be screening for multiple cancers					
Other (please specify)					

15. Which of the following would make you most willing to receive a blood-based multi-cancer early detection and screening test? Please rate the following, with 1 being not at all convincing and 5 being extremely convincing.

	**1 Not at all convincing**	**2 Slightly convincing**	**3 Moderately convincing**	**4 Very convincing**	**5 Extremely convincing**
Discussion with my healthcare provider					
Discussion with family/friends					
Educational material from a doctor's office					
Educational material from your employer (email, brochure, mail)					

Educational material from your insurance company (email, brochure, mail)	
Educational materials from a testing company (email, brochure, mail)	
Advocacy organizations' websites	
TV / radio /podcast commercials	
Social media ads	
Online ads	
Other (please specify)	

16. What type of support from your healthcare provider would impact your willingness to receive a blood-based multi-cancer early detection and screening test? *Please select all that apply*
    a. Time spent discussing the pros and cons of the test
    b. Time spent answering my questions about the test
    c. Help navigating insurance coverage of the test
    d. Educational materials about the test
    e. A follow-up call or appointment discussing the results of the test
    f. Resources for positive test results
    g. Help scheduling any follow up appointments needed for positive test results
    h. Other (please specify)
